# Entwicklung und Fortschritte des Nationalen Obduktionsnetzwerks (NATON)

**DOI:** 10.1007/s00292-024-01307-8

**Published:** 2024-03-01

**Authors:** Svenja Windeck, Kristina Allgoewer, Saskia von Stillfried, Lucas Triefenbach, Ulrike Nienaber, Roman David Bülow, Rainer Röhrig, Benjamin Ondruschka, Peter Boor, NATON

**Affiliations:** 1 Institut für Pathologie, Universitätsklinikum RWTH Aachen, Pauwelsstraße 30, Aachen, Deutschland; 2 Institut für Rechtsmedizin, Universitätsklinikum Hamburg-Eppendorf, Hamburg, Deutschland; 3 Institut für Medizinische Informatik, Universitätsklinikum RWTH Aachen, Pauwelsstraße 30, Aachen, Deutschland

**Keywords:** Autopsie, COVID-19, Krankheitsmanagement, Deutschland, Register, Autopsy, COVID-19, Disease management, Germany, Registries

## Abstract

**Hintergrund:**

Obduktionen gelten seit langem als der Goldstandard für die Qualitätssicherung in der Medizin. Die COVID-19-Pandemie hat ihr Potenzial für das Verständnis der Pathophysiologie, Therapie und Krankheitsbewältigung wieder in den Fokus gerückt. Im April 2020 wurde das Deutsche Register für COVID-19-Obduktionen (DeRegCOVID) eingerichtet, gefolgt vom Konsortium DEFEAT PANDEMIcs (2020–2021), das sich zum Nationalen Obduktionsnetzwerk (NATON) entwickelte.

**DeRegCOVID:**

DeRegCOVID sammelte und analysierte über 3 Jahre hinweg Obduktionsdaten von COVID-19-Verstorbenen in Deutschland und ist damit die größte nationale multizentrische Obduktionsstudie. Die Ergebnisse identifizierten entscheidende Faktoren für schwere/tödliche Fälle, wie z. B. pulmonale vaskuläre Thromboembolien, und das komplizierte Zusammenspiel von Virus und Immunsystem. DeRegCOVID diente als zentraler Hub für die Datenanalyse, Forschungsanfragen und öffentliche Kommunikation und spielte eine wichtige Rolle im Austausch mit Politik und öffentlichem Gesundheitswesen.

**NATON:**

NATON wurde vom Netzwerk Universitätsmedizin (NUM) initiiert und entwickelte sich zu einer nachhaltigen Infrastruktur für autopsiebasierte Forschung. Ziel ist die Bereitstellung einer Daten- und Methodenplattform, die die Zusammenarbeit zwischen Pathologie, Neuropathologie und Rechtsmedizin fördert. Die Struktur unterstützt eine rasche Rückkopplung zwischen Forschung, Patientenversorgung und Pandemiemanagement.

**Schlussfolgerung:**

DeRegCOVID hat wesentlich zum Verständnis der COVID-19-Pathophysiologie beigetragen. Durch seinen modularen Aufbau will das Nationale Obduktionsregister (NAREG) nun die Zusammenarbeit auf nationaler sowie internationaler Ebene weiter verbessern.

## Abstract (en)

**Background:**

Autopsies have long been considered the gold standard for quality assurance in medicine, yet their significance in basic research has been relatively overlooked. The COVID-19 pandemic underscored the potential of autopsies in understanding pathophysiology, therapy, and disease management. In response, the German Registry for COVID-19 Autopsies (DeRegCOVID) was established in April 2020, followed by the DEFEAT PANDEMIcs consortium (2020–2021), which evolved into the National Autopsy Network (NATON).

**DeRegCOVID:**

DeRegCOVID collected and analyzed autopsy data from COVID-19 deceased in Germany over three years, serving as the largest national multicenter autopsy study. Results identified crucial factors in severe/fatal cases, such as pulmonary vascular thromboemboli and the intricate virus-immune interplay. DeRegCOVID served as a central hub for data analysis, research inquiries, and public communication, playing a vital role in informing policy changes and responding to health authorities.

**NATON:**

Initiated by the Network University Medicine (NUM), NATON emerged as a sustainable infrastructure for autopsy-based research. NATON aims to provide a data and method platform, fostering collaboration across pathology, neuropathology, and legal medicine. Its structure supports a swift feedback loop between research, patient care, and pandemic management.

**Conclusion:**

DeRegCOVID has significantly contributed to understanding COVID-19 pathophysiology, leading to the establishment of NATON. The National Autopsy Registry (NAREG), as its successor, embodies a modular and adaptable approach, aiming to enhance autopsy-based research collaboration nationally and, potentially, internationally.

Seit jeher gelten Obduktionen als ein wichtiges Instrument zum Verständnis von Krankheiten. Nicht zuletzt die COVID-19-Pandemie hat ihren hohen Wert und ihr großes Potenzial deutlich gemacht. Seit April 2020 haben sich hierzu erstmals Pathologie, Neuropathologie und Rechtsmedizin zum Deutschen Register für COVID-19 Obduktionen (DeRegCOVID) zusammengeschlossen. Aus dem erfolgreichen Register wuchs schnell das Deutsche Forschungsnetz für Obduktionen bei Pandemien (DEFEAT PANDEMIcs), das sich zum Nationalen Obduktionsnetzwerk (NATON) weiterentwickelt hat. In DeRegCOVID sowie NATON sind bislang 33 universitäre sowie 6 nichtuniversitäre Zentren beteiligt, darunter seit 2023 erstmals auch zwei internationale Zentren aus Österreich.

## Hintergrund

Obduktionen gelten seit jeher als Goldstandard zur Qualitätssicherung in der Medizin. Dagegen wurde die Bedeutung von Obduktionen für die Grundlagenforschung als weniger relevant wahrgenommen. Die COVID-19-Pandemie hat jedoch deutlich gezeigt, dass Obduktionen für das Verständnis der Pathophysiologie und somit auch für die Therapie und das Management von Erkrankungen großes Potenzial haben. Aus diesem Grund sind eine rasche Erfassung von Obduktionsdaten und der interdisziplinäre Austausch über Krankheitsverläufe und Auswirkungen der Infektion von höchster Bedeutung. Zu Beginn der COVID-19-Pandemie entstand somit im April 2020 die Idee, Obduktionen SARS-CoV-2-positiver Patientinnen und Patienten systematisch zu erfassen, um Erkenntnisse über die Auswirkungen des Virus auf Zellen und Gewebe zu gewinnen. Entwickelt wurde dazu das Deutsche Register für COVID-19 Obduktionen (DeRegCOVID), das seitdem Zentren aus Pathologie, Neuropathologie und Rechtsmedizin von mittlerweile 33 universitären sowie 6 nichtuniversitären Standorten vernetzt hat. Die gemeinsame Obduktionsforschung zeigte die entscheidende Rolle von pulmonalen (mikro-)vaskulären Thromboembolien, eine systemische Virusausbreitung und das komplexe Wechselspiel zwischen Viren und dem Immunsystem bei schweren COVID-19-Erkrankungen und tödlichen Verläufen. Sie hat somit gezeigt, wie wertvoll eine vernetzte und interdisziplinäre Obduktionsforschung auch für die Grundlagenforschung sein kann.

Im Folgenden wird aufgezeigt, welche Bedeutung das DeRegCOVID und das darauf aufbauende Netzwerk bei der Erforschung der Auswirkungen von COVID-19 gespielt haben. Außerdem soll ein besonderer Fokus auf die neuesten Entwicklungen hin zu einem entitätenübergreifenden nationalen Obduktionsregister gelegt werden.

## Ein COVID-19-Obduktionsregister

**Figure 1:**
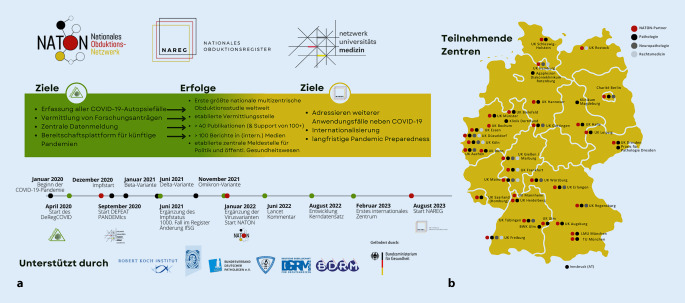

Das DeRegCOVID hatte sich 4 übergeordnete Ziele gestellt:

1. Zentrale Erfassung möglichst aller COVID-19-Obduktionen in Deutschland (s. Abschn. „Ziel 1“),
2. Unterstützung der teilnehmenden Zentren und Forschenden (s. Abschn. „Ziel 2“),
3. zentrale Datenauswertung und Meldung (s. Abschn. „Ziel 3“),
4. Etablierung als zentrale Vermittlungsstelle und Kommunikationshub (s. Abschn. „Ziel 4“, Abb. 1).

### Ziel 1.

Das Hauptziel des DeRegCOVID bestand darin, zentral systematisch Daten möglichst aller Obduktionen von COVID-19-Verstorbenen in Deutschland zu sammeln und zu analysieren und somit multizentrische, obduktionsgetriebene Forschung zu ermöglichen. Dabei wurden 2 grundlegende Prinzipien etabliert: 1. Alle Proben werden dezentral im jeweiligen Obduktionszentrum verwahrt. 2. Alle Daten bleiben im Besitz des jeweiligen Zentrums und können lokal unabhängig genutzt und publiziert werden. Während einer Laufzeit von knapp 3 Jahren konnten insgesamt über 2150 Datensätze sowie Informationen zu über 59.000 Bioproben von 33 universitären sowie 6 nichtuniversitären Zentren in Deutschland gesammelt werden. Im Jahr 2023 folgten die ersten beiden internationalen Zentren aus Österreich. Mit Hilfe aller Zentren war es möglich, aus dem DeRegCOVID die erste und bislang größte nationale multizentrische COVID-19-Obduktionsstudie weltweit zu generieren und zu publizieren [1]. Eine weitere Auswertung der Registerdaten zeigte, dass ECMO („extracorporeal membrane oxygenation“) hauptsächlich bei jüngeren männlichen COVID-19-Patienten angewendet wurde und in COVID-19-Fällen im Vergleich zu Non-COVID-19-Fällen mit einem höheren Risiko für Blutungen, insbesondere intrakranielle Blutungen, verbunden war. Intrakranielle Blutungen waren bei ECMO-Patienten eine häufige Todesursache [2]. Aktuell ist eine weitere gemeinsame Studie, eine Auswertung der Registerdaten zu Obduktionen nach SARS-CoV-2-Impfung, in Vorbereitung. Dabei werden alle an der Gewinnung der analysierten Registerdaten beteiligten Personen, sofern gewünscht, als Collaborator oder Koautoren (anhand der konsentierten Publikationsordnung abhängig von der Anzahl an gemeldeten Obduktionen für das Netzwerk) an allen Studien beteiligt.

### Ziel 2.

Darüber hinaus fungierte das Register seit Beginn als Vermittlungsstelle zwischen Obduktionszentren und Forschenden. Durch diese Aufgabe konnte das DeRegCOVID in vergleichsweiser kurzer Zeit über 34 Publikationen in medizinischen Fachzeitschriften unterstützen. Zum Beispiel wurden Forschungsergebnisse, die zu Pandemiebeginn vor dem funktionellen Start des Registers entstanden waren, wie Gefäßveränderungen (z. B. Endotheliitis, Mikroangiopathie und aberrante Angiogenese) detaillierter untersucht [3–6]. Auf zellulärer Ebene wurden vaskuläre neutrophile Inflammation und Immunothrombose und die T‑Zell-Dichte in kranialen Nerven in COVID-19 charakterisiert [7, 8]. Durch Untersuchungen an durch Obduktionen gewonnenen Geweben konnte bestätigt werden, dass zytotoxische T‑Zellen sowie profibrotische Makrophagen bei schweren COVID-19-Verläufen vermehrt nachweisbar sind [9, 10]. Außerdem konnte SARS-CoV-2-RNA in allen Organen nachgewiesen werden [11, 12].

### Ziel 3.

Das Register sollte auch als zentrale Vermittlungsstelle für Datenanalyse und Forschungsanfragen dienen. Die Möglichkeit, flexibel auf aktuelle Entwicklungen der Pandemie reagieren zu können, stand von vornherein im Fokus des Projekts. Deswegen wurde das Register technisch wie inhaltlich an die sich neu entwickelnden Situationen adaptiert und erweitert, z. B. mit der Ergänzung des Impfstatus und Angaben zu relevanten Virusvarianten, Umstellung des Logins auf eine sicherere 2‑Faktor-Authentifizierung und einem Systemumzug zu einem anderen elektronischen Datenerfassungssystem. Zur Unterstützung der Obduktionszentren wurde zudem bereits zu Anfang der Pandemie die erste Empfehlung zur Durchführung von infektiösen COVID-19-Obduktionen durch das DeRegCOVID ausgearbeitet und allen obduzierenden Zentren zur Verfügung gestellt [13].

### Ziel 4.

Seit Beginn der Pandemie unterstützte das DeRegCOVID außerdem die Kommunikation mit der Öffentlichkeit, Politik sowie dem öffentlichen Gesundheitswesen. In 2021 konnte auf Grundlage eines intensiven Austausches mit dem Bundesministerium für Gesundheit (BMG) das Infektionsschutzgesetz (§§ 25, 60 IfSG) zur Erhöhung von durch Gesundheitsämter angeordneten Obduktionen angepasst werden. Ferner stand das DeRegCOVID sowohl Politik als auch dem Robert Koch-Institut (RKI) und BMG bei Fragen stets zur Verfügung und hat wiederholt (zum Teil in kürzester Zeit) auf Anfragen des RKI und BMG relevante Daten geliefert. Die Daten dienen weiterhin auch der Politik, z. B. dokumentiert durch die Publikation des Wissenschaftlichen Dienstes des Bundestages [15].

Eine enge Zusammenarbeit mit Presse und Öffentlichkeit, die zum großen Teil durch jeweilige Beteiligte durchgeführt worden ist, führte zu einer regen Berichterstattung über das Register. Seit 2020 wurden somit über 100 Beiträge und Artikel in nationalen sowie internationalen Radio‑, TV-, Print- und Onlinemedien veröffentlicht. In diesen werden die Relevanz und die Ergebnisse obduktionsgestützter COVID-19-Forschung laienverständlich thematisiert, um die breite Öffentlichkeit über die Entwicklungen des Registers und die Relevanz von Obduktionen, aber auch die Auswirkungen von SARS-CoV‑2 zu informieren.

Befürwortet und unterstützt wurde das Vorhaben bereits zu Beginn durch den Bundesverband Deutscher Pathologen (BDP) und die Deutsche Gesellschaft für Pathologie (DGP). Der Erfolg des DeRegCOVID führte rasch zu einer Kooperation mit der Deutschen Gesellschaft für Neuropathologie und Neuroanatomie (DGNN) und der Fusionierung ihres Registers (CNS-COVID-19) mit dem DeRegCOVID. Zuletzt gelang der Einschluss des Fachbereichs Rechtsmedizin, wodurch das Register jetzt zusätzlich vom Berufsverband Deutscher Rechtsmediziner (BDRM) und der Deutschen Gesellschaft für Rechtsmedizin (DGRM) unterstützt wird. Über die 5 Fachgesellschaften hinaus haben sich auch das Robert Koch-Institut (RKI), das Paul-Ehrlich-Institut (PEI) und das Bernhard-Nocht-Institut für Tropenmedizin (BNITM) für eine Zusammenarbeit mit dem Register ausgesprochen.

Infolge der Veröffentlichung des ersten Reports des DeRegCOVID wurde in der Zeitschrift *The Lancet Regional Health – Europe* ein unabhängiger Kommentar veröffentlicht, in dem das Register als „Beispiel für Europa und die Welt“ hervorgehoben wurde [14].

## Der Aufbau eines deutschlandweiten Obduktionsnetzwerks

Wenige Monate nach Start der Pandemie wurde die Initiative „Netzwerk Universitätsmedizin“ (NUM) ins Leben gerufen. Initial hat sich das NUM als Teil des Krisenmanagements zur Aufgabe gemacht, die COVID-19-Forschung an deutschen Universitätskliniken zu bündeln und besser zu koordinieren. Mittlerweile wird an einer Verstetigung des NUM über die COVID-19-Pandemie hinaus gearbeitet, um die vernetzte Forschung in Deutschland weiter zu verbessern und ein flächendeckendes *Pandemic-Preparedness-*Netz zu etablieren.

In der initialen Förderphase des NUM wurde auf Basis des Registers das Konsortium DEFEAT PANDEMIcs (Deutsches Forschungsnetzwerk Autopsien bei Pandemien) eingerichtet. Das Ziel des Projekts bestand im Aufbau eines deutschlandweiten Obduktionsnetzwerks für den Pandemiefall, um systematisch und strukturiert Daten, Bioproben und Erkenntnisse möglichst vollständig, umfassend und zeitnah zu erfassen, zusammenzuführen und den Netzwerkpartnern zur Auswertung zur Verfügung zu stellen. Der wertvolle Beitrag von DEFEAT PANDEMIcs zur Bewältigung der COVID-19-Pandemie diente als Katalysator für ein multimodulares Nationales Obduktionsnetzwerk (NATON).

Das allgemeine Ziel von NATON ist es nun, eine expertenbetriebene Daten- und Methodenplattform für vernetzte obduktionsgestützte Forschung innerhalb des NUM bereitzustellen. Die wichtigsten strukturellen Ziele von NATON liegen in der (1) Bereitstellung einer langfristigen Infrastruktur und von Services, die durch die Community vorangetrieben werden, (2) der Vernetzung der Expertise von Pathologie, Neuropathologie und Rechtsmedizin zur kollaborativen Obduktionsprobengewinnung und -aufarbeitung sowie (3) der Bereitstellung einer zentralen Informationsaustauschstelle für Teilprojekte der NUM-Förderlinien sowie für externe Stakeholder, einschließlich des öffentlichen Gesundheitswesens.

Durch etablierte Strukturen und die Experten- und Kompetenzbündelung obduktionsbasierter Forschung wird eine rasche Rückkopplung zwischen Forschung, Patientenversorgung und Pandemiemanagement ermöglicht. Die Integration von NATON in das NUM ermöglicht es dabei, hochgradig komplementäres Fachwissen und Ressourcen im Bereich Pathologie, Neuropathologie und Rechtsmedizin für die Obduktionsforschung zur Verfügung zu stellen, während NATON gleichzeitig von den bereits verfügbaren und etablierten Rahmenbedingungen, der Infrastruktur und dem Fachwissen innerhalb von NUM profitieren kann.

Auch nach der COVID-19-Pandemie kann NATON auf vielfältige Weise zur *Pandemic Preparedness *in Deutschland beitragen. Die kontinuierliche Überwachung von Todesfällen auf Hinweise zu neuartigen Erkrankungen oder die Häufung von Symptomen und deren Meldung an die Gesundheitsbehörden ermöglichen eine frühzeitige Erkennung von Ausbrüchen. Im Pandemiefall kann die Pathophysiologie neuer Erreger durch systematische Obduktionen schnell charakterisiert werden. Langfristig könnte NATON als Beratungsinstanz für Obduktionszentren, das Bestattungswesen und Gesundheitsbehörden zum Umgang mit infektiösen Verstorbenen zur Verfügung stehen. Im weiteren Verlauf einer zukünftigen Pandemie könnten vulnerable Bevölkerungsgruppen identifiziert und Todesfälle systematisch auf neuartige Infektionserreger oder auf neuartige Erregervarianten untersucht werden (auch retrospektiv, da Gewebeproben langzeitarchiviert werden), therapeutische Interventionen evaluiert, Todesfälle im zeitlichen Zusammenhang mit Impfungen analysiert sowie Gewebeveränderungen bei Verstorbenen mit Langzeiterkrankungen dokumentiert und detailliert analysiert werden.

**Figure 2:**
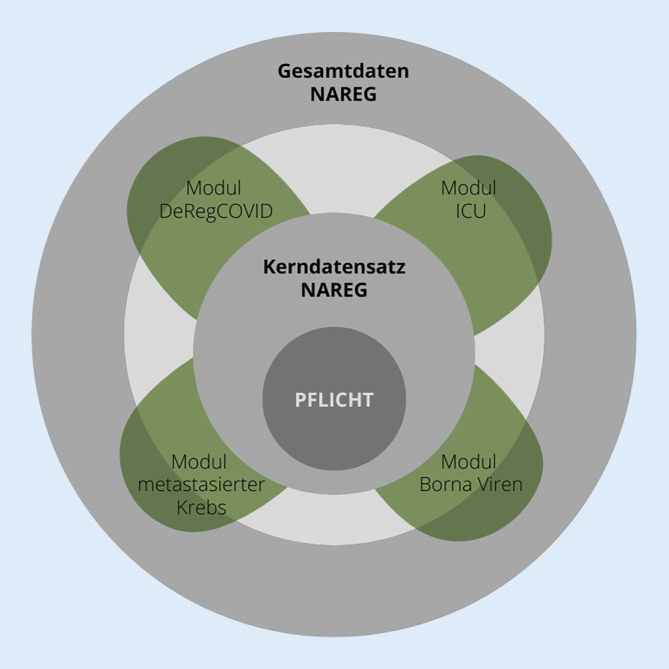

NATON ist nicht nur für Infektionserkrankungen, sondern auch für andere bzw. prinzipiell alle obduktionsgetriebenen Projekte und Anwendungsfälle konzipiert. Um ebendiese Anwendungsfälle jenseits von COVID-19 einschließen zu können, wird das elektronische Rückgrat von NATON, das DeRegCOVID, zu NAREG (Nationales Obduktionsregister) transferiert. NAREG basiert dabei auf einem modularen Aufbau, der anwendungsfallspezifisch angepasst und skaliert werden kann (siehe Abb. 2). Das Herzstück von NAREG ist der in Zusammenarbeit mit dem BDP entwickelte Kerndatensatz Obduktion, der unterschiedliche Angaben beinhaltet, z. B. autoptische Todesursachen und Befunde, klinische Angaben, klinische Todesursachen usw. Er schafft die Grundlage dafür, zukünftig potenziell alle routinemäßig durchgeführten Obduktionen standardisiert zu erfassen. Der Kerndatensatz wird um forschungsprojektspezifische Aufbaumodule erweitert. Die Daten, die in den Aufbaumodulen erhoben werden sollen, können je nach Bedarf des jeweiligen Forschungsprojektes (z. B. metastasierte Krebserkrankungen, fatale infektiöse Erkrankungen usw.) spezifisch angepasst werden. Ein erster Use Case zur Bornavirus-Enzephalitis wurde bereits in Kooperation mit dem Paul-Ehrlich-Institut und dem Bernhard-Nocht-Institut für Tropenmedizin angegangen. Für jedes Aufbaumodul wird es ein entsprechendes Amendment zum Registerprotokoll geben.

## Eine nachhaltige Infrastruktur

Innerhalb von NATON gewährleistet das NAREG den Betrieb der IT-Basisfunktionalität und damit die kontinuierliche Anpassungsfähigkeit an neue Gegebenheiten. Dazu zählen der Support für NATON-Partner und externe Forschende zur stetigen Optimierung der Benutzerfreundlichkeit, die Sicherstellung von Datensicherheit und Datentransfer sowie die Kuratierung, Standardisierung und Harmonisierung der Daten. Zur Förderung der Standardisierung von obduktionsspezifischen Daten wurde der oben erwähnte Kerndatensatz Obduktion erarbeitet. Dieser orientiert sich an der 3. Auflage der *S1-Leitlinie zur Durchführung von Obduktionen in der Pathologie* [16]. Der Kerndatensatz ermöglicht einen modularen Aufbau des Registers. NAREG soll nicht nur *Pandemic Preparedness* unterstützen, sondern auch die Grundlage dafür schaffen, multizentrische obduktionsgetriebene Forschungsprojekte sowie qualitätssichernde Maßnahmen an Krankenhäusern zu etablieren.

Die Methodenplattform des NATON prüft die fachliche Durchführbarkeit von externen und internen Forschungsprojekten und -anfragen sowie deren Qualität durch die Integration der organspezifischen Expertisen der beteiligten Expertinnen und Experten. Dies ist essenziell, da die Erfahrung mit postmortalen Proben und den damit einhergehenden Besonderheiten unter vielen Forschenden gering ist. Innerhalb der Methodenplattform wird ferner organspezifische Diagnostik bei komplexen Sterbefällen angeboten und durchgeführt und es werden fortlaufend diagnostische Kriterien und Methoden zur validierten Befunderstellung erarbeitet. Zur Sicherstellung der bestmöglichen Qualität und Harmonisierung von Obduktionsproben werden Empfehlungen zur Verbesserung der Probenqualität und -entnahme erarbeitet. Um höhere Obduktionszahlen zu generieren, sollen die lokalen Obduktionszentren weiter unterstützt und aktives *Capacity Building* in den Fachgesellschaften praktiziert werden. Im Bemühen, aktuelle Entwicklungen und Veränderungen an relevante Stakeholder des öffentlichen Gesundheitswesens zeitnah melden zu können, werden die in NAREG eingehenden Daten beobachtet und Surveillanceindikatoren genutzt.

Verbunden und koordiniert wird NATON durch das NATON Office, das sowohl für die interne Koordination als auch für die externe Kommunikation zuständig ist und in ständigem Kontakt mit Presse, Politik und dem öffentlichen Gesundheitswesen steht.

### Infobox Die Technik hinter dem Register

Wie auch bei seinem Vorgänger DeRegCOVID handelt es sich bei NAREG um ein zentrales Register, welches in einem ISO-27001-zertifizierten Rechenzentrum betrieben wird. Benutzer erhalten einen webbasierten Zugang, der durch eine 2‑Faktor-Authentifizierung gesichert ist. Die Datenübertragung erfolgt über eine TLS-verschlüsselte Verbindung. Als elektronisches Datenerfassungssystem kommt REDCap zum Einsatz.

## Künftige Herausforderungen und Perspektiven

Die Ausweitung des DeRegCOVID auf NAREG birgt neben vielen Möglichkeiten auch diverse Herausforderungen. So sind für den Einschluss möglichst aller Obduktionen in Deutschland ethische und technische Herausforderungen zu meistern.

Ein Beispiel ist die unklare Rechtslage in Bezug auf den Schutz personenbezogener Daten von Verstorbenen, die nicht unter die allgemeine Datenschutz-Grundverordnung (DSGVO) fallen. Diese muss in Zukunft für einen erfolgreichen Registerbetrieb geklärt werden. Eine Einwilligung der Verstorbenen – sowohl in die Obduktion als auch in die Eintragung dieser in das NAREG – sind nach dem Tod nicht mehr möglich. Dieser Sachverhalt fällt nicht eindeutig unter die allgemeine DSGVO, sodass eine, bundesweit gesehen, unklare Rechtslage in Bezug auf den Schutz personenbezogener Daten von Verstorbenen resultiert. Eine Lösungsmöglichkeit ist die Integration der Einwilligung in die Verarbeitung der Autopsiedaten in den *Broad Consent* der Medizininformatik-Initiative. Allerdings werden dadurch weder die Autopsien in nichtuniversitären Pathologien noch in klinikunabhängigen pathologischen Instituten erfasst. Auch in Universitätskliniken liegt dann keine Einwilligung bei externen Zuweisungen vor. Daher ist aus wissenschaftlicher Sicht vom Gesetzgeber eine Opt-out-Lösung unter Nutzung der Forschungsklauseln in der DSGVO zu schaffen. Totensorgeberechtigte Angehörige können einer klinischen Obduktion zustimmen, wenn dies ihrem freien Willen und dem mutmaßlichen Willen der verstorbenen Person entspricht. Ferner sind pathologische und neuropathologische Obduktionen noch immer überwiegend Individualentscheidungen der behandelnden Ärztinnen und Ärzte, sodass der Aus- und Fortbildung der klinischen Kolleg*innen bezüglich des Wertes der Obduktionen zur Qualitätssicherung, Behandlungsoptimierung und dem wissenschaftlichen Zugewinn eine wichtige Rolle zukommt. (Meta‑)Analysen zeigen, dass Obduktionen nach wie vor unerwartete, klinisch relevante und outcomerelevante Diagnosen in ca. 10 % der Obduktionsfälle aufdecken können [17–19]. Folgerichtig sollte, wie nun im Infektionsschutzgesetz (IfSG) geschehen, die Verankerung von Obduktionen als hilfreiches Tool in medizinischer Forschung und Qualitätssicherung ausgeweitet werden. Eine Bewertung von Obduktionen als Qualitätsindikator für Krankenhäuser wäre sinnvoll.

Hierfür sind einheitliche Dokumentations- und Kommunikationsstandards notwendig. Die NAREG-Infrastruktur bietet durch ihren modularen, individuell anpassbaren Aufbau in Zukunft die Möglichkeit der Schnittstellenbildung zu anderen, bereits vorhandenen sowie neuen Applikationen, Strukturen und Registern. Dieser Schlüsselfaktor ermöglicht die langfristige Vision, von der händischen Eintragung der Obduktionen ins Register auf eine vollautomatisierte Eintragung umzustellen. Die Annotation ist hierbei eine wesentliche technische Hürde, da gerade bei autopsiespezifischen Daten, wie bspw. der Lagerung von Leichen, bisher semantische Annotationen fehlen, welche aufwendig händisch verknüpft werden müssen.

Der erfolgreiche fachbereichsübergreifende Zusammenschluss von Obduktionszentren in Deutschland lässt die Perspektive zu, in Zukunft eine Ausweitung des NAREG über die Bundesgrenzen hinaus zu erreichen. Im DeRegCOVID konnten bereits zwei österreichische Zentren (beide in Innsbruck) gewonnen werden. Weitere Gespräche, z. B. in Österreich oder der Schweiz, erlauben die Zukunftsvision eines europäischen oder internationalen Obduktionsregisters. Hierbei könnte ein übergeordnetes NAREG-Dashboard eine Datenübersicht und -meldung an Politik und Entscheidungsträger sowie die Dissemination in die internationale Fachcommunity gewährleisten und die erfolgreiche, technische Infrastruktur des NAREG bereitstellen.

## Schlussfolgerungen

Während seiner knapp dreijährigen Laufzeit trug DeRegCOVID maßgeblich dazu bei, unser Verständnis der Pathophysiologie und der klinischen Verläufe von COVID-19 zu vertiefen und diente als Infrastruktur für die Etablierung einer nachhaltigen *Pandemic-Preparedness*-Struktur in Form des Nationalen Obduktionsnetzwerks. Die Tatsache, dass Standorte aus Ländern bzw. Institutionen, in denen die Obduktion traditionell einen hohen Stellenwert hat, trotz des Fehlens einer Registerinfrastruktur ebenfalls schnell zu bedeutenden Ergebnissen, wenngleich an kleineren Fallzahlen kamen [20, 21], demonstriert das große Potenzial international vernetzter, obduktionsgestützter Forschung. Mit dem Nachfolger des Registers – NAREG – werden gemeinsam mit NATON die Bemühungen fortgesetzt, die kontinuierliche Zusammenarbeit auch über die Pandemie hinaus aufrechtzuerhalten und die (inter-)nationale obduktionsbasierte Forschung entitätenübergreifend weiter zu verbessern.

Bei Interesse an einer Kooperation bzw. an der Teilnahme an NATON und NAREG sind unter https://naton.network/ weitere Informationen und Kontaktmöglichkeiten abrufbar.

## Fazit für die Praxis

- Das Nationale Obduktionsnetzwerk (NATON) sowie das Deutsche Register für COVID-19 Obduktionen (DeRegCOVID) unterstützen obduktionsgetriebene multizentrische Forschung.
- Das Register sowie die teilnehmenden Zentren haben maßgeblich dazu beigetragen, das Verständnis der Pathophysiologie und der Verläufe von COVID-19 zu vertiefen, und dabei den hohen Wert vernetzter, obduktionsbasierter Forschung betont.
- Mit dem Nationalen Obduktionsregister (NAREG) soll die erfolgreiche Arbeit des DeRegCOVID für weitere Anwendungsfälle über COVID-19 hinaus fortgeführt werden. Das wird durch den modularen Aufbau von NAREG ermöglicht. NAREG soll ferner die Zusammenarbeit in der obduktionsbasierten Forschung national sowie international weiter fördern.
- NATON sowie NAREG sind offen für alle interessierten Zentren.
